# Postoperative Horner’s syndrome after video-assisted thyroidectomy: a report of two cases

**DOI:** 10.1186/1477-7819-11-315

**Published:** 2013-12-30

**Authors:** Xin Ying, Guan Dandan, Chen Bin

**Affiliations:** 1 Thyroid and Breast Surgery Department, Zhejiang Provincial People’s Hospital, Shangtang Rd 158#, Hangzhou, China; 2 General Surgery Department, Hangzhou First People’s Hospital, Huansha Rd 261#, Hangzhou, China

**Keywords:** Thyroidectomy, Horner’s syndrome, Etiology

## Abstract

Horner’s syndrome is a rare complication after video-assisted thyroidectomy (VAT). We present two cases of thyroid microcarcinoma who presented with Horner’s syndrome on day 2 after surgery. Ultrasonography showed no hematoma or fluid collection in the surgical field. For the first case, the symptoms were much relieved 4 months later. However, the right pupil remained smaller. For the second case, the symptoms were relieved 3 days later. The symptoms were caused by local trauma to the sympathetic chain and likely occurred during retraction of the carotid sheath. Surgeons should be aware of the possibility of Horner’s syndrome after VAT.

## Background

Oculosympathetic paresis was named after Friedrich Horner, a Swiss ophthalmologist [1] or Claude Bernard, a French physiologist [2]. Thus, it was referred as Claude Bernard Horner’s syndrome or Horner’s syndrome. Horner’s syndrome, which is characterized by ipsilateral miosis, ptosis, enophthalmos, and facial anhidrosis, is a rare complication following conventional thyroid surgery [3,4]. We report two cases after video-assisted thyroidectomy (VAT).

## Case presentation

Case one: A 34-year-old female was found to have a right thyroid mass of 0.4 x 0.5 x 0.4 mm. Ultrasonography demonstrated the mass was hypoechoic and irregular. No obvious lymph node metastasis was shown in the neck. The preoperative fine needle aspiration disclosed papillary thyroid cancer. The patient underwent video-assisted right thyroidectomy, isthmusectomy and right central neck dissection with a 2.5-cm incision on the neck.

**Figure 1:**
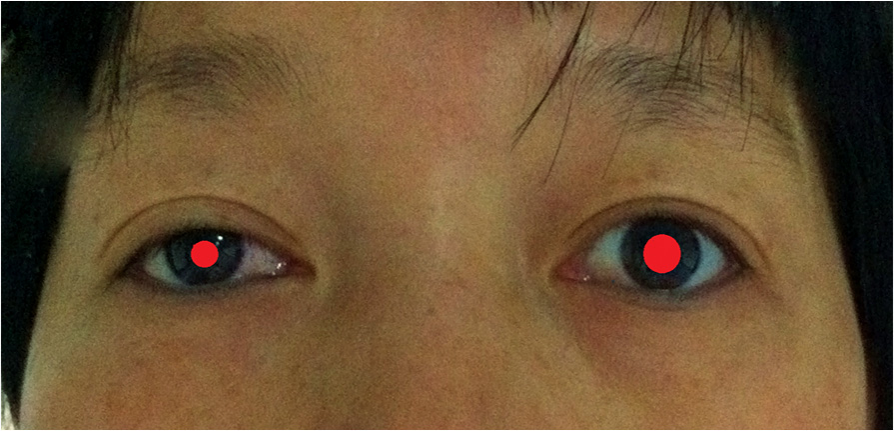
**Horner’s syndrome (2 days post surgery).** Miosis, eyelid ptosis and enophthalmos were noted on the right side.

**Figure 2:**
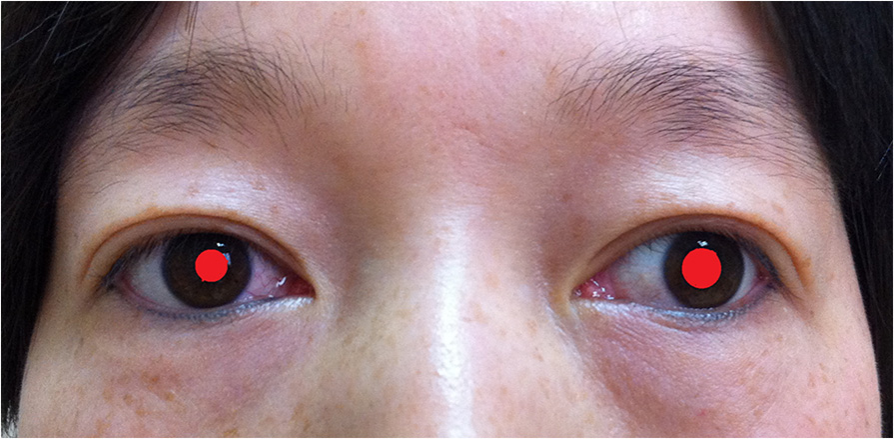
Horner’s syndrome (4 months post surgery).

On day 2 after surgery, the patient presented with miosis and eyelid ptosis (Figure 1). She had no anhidrosis or vascular dilatation of the lateral part of the face. Ultrasonography ruled out the possibility of hematoma or fluid collection in the surgical field. Steroid tablets were given orally for 4 days. The postoperative pathological result confirmed right thyroid papillary carcinoma with no lymph node metastasis. During 4 months of follow-up, many of the patient’s symptoms of Horner’s syndrome were relieved without treatment (Figure 2). However, the right pupil was still smaller than the size preoperatively.

Case two: A 24-year-old female was diagnosed with a microcarcinoma of the left thyroid, measuring approximately 0.5 × 0.6 × 0.3 mm. Preoperative fine needle aspiration demonstrated a papillary carcinoma. Video-assisted left thyroidectomy, isthmusectomy and left central neck dissection were performed. By the second day after the operation, the patient had developed Horner’s syndrome. However, it was relieved 3 days later without any medical intervention.

## Discussion

The cervical sympathetic chain (CSC) is located posteromedially to the carotid sheath, anterior to the longus muscles and under the prevertebral fascia [5]. The cervical sympathetic trunk (CST) can pass within the posterior wall of the carotid sheath. Hence, compression of the carotid sheath by the retractor is a possible cause of CST injury during thyroidectomy. The CSC is composed of three ganglia: the superior, intermediate and inferior. The superior ganglion, which is normally the largest of the CSC ganglia, is located between the levels C2 to C4 of the vertebral column. The intermediate ganglion of the CST is the smallest. The inferior thyroid artery may cross the intermediate ganglion anteriorly or posteriorly. If the middle ganglion and inferior thyroid artery are too close together, the middle ganglion can be injured during the ligation of the inferior thyroid artery. This may be another reason for postoperative Horner’s syndrome after thyroidectomy.

Now, VAT is an alternative to conventional surgery due to its minimal invasiveness. In VAT, however, the small incision on the neck may need to be retracted more forcefully in order to expose the carotid sheath laterally. Theoretically, this may lead to higher incidences of postoperative Horner’s syndrome. The incidence of Horner’s syndrome was around 0.2% after conventional surgery [3]. However, until recently, the incidence of Horner’s syndrome was largely unknown after VAT. From 2011 to 2012, 537 cases of VAT were performed in our department. Two cases of postoperative Horner’s syndrome occurred (0.4%). According to the literature, [6,7] there were no statistically significant differences between VAT and conventional thyroidectomy for complications, such as transient recurrent laryngeal nerve palsy and hypoparathyroidism. In our study, the incidence of postoperative Horner’s syndrome was relatively higher in VAT. However, it might be due to a small sample or short learning curve. Further data is required for confirmation.

To the best of our knowledge, these are the first cases reported for Horner’s syndrome after VAT. The patient’s heart beat once dropped to 40 bpm in surgery for the first case. For the second case, Horner’s syndrome was relieved spontaneously 5 days after surgery. We highly suspect that the symptoms of the two cases were caused by trauma to the sympathetic chain during retraction of the carotid sheath. The difference was that the first case was more severe than the second one. For the first case, ischemia of the CST attributed to retraction was suspected, which took 4 months to recover. Thus, surgeons should pay attention to avoid lateral overexposure during VAT.

The literature has suggested that other possible causes of damage to the CSC could be due to anatomical variations, ischemia or compression caused by hematoma to the sympathetic chain [8-10].

## Conclusions

VAT could lead to postoperative Horner’s syndrome. Patients should be informed of this complication before surgery.

## Consent

Written informed consent was obtained from patients for publication of this case report and all accompanying images. A copy of the written consent is available for review by the Editor-in-chief of this journal.

## Abbreviations

CSC: Cervical sympathetic chain; CST: Cervical sympathetic trunk; FNA: Fine needle aspiration; VAT: Video-assisted thyroidectomy.

## Competing interests

The authors declare no competing interests.

## Authors’ contributions

XY drafted the manuscript and searched the literature. CB and GDD were involved in the treatment of the patients. CB reported the pathological findings and prepared the photographs. All authors have read and approved the final manuscript.

## References

[B1] van der Wiel HL Johann Friedrich Horner (1831–1886) J Neurol 2002 11 636 637 10.1007/s004150200079 12021960

[B2] Ross IB The role of Claude Bernard and others in the discovery of Horner’s syndrome J Am Coll Surg 2004 11 976 980 10.1016/j.jamcollsurg.2004.06.024 15555981

[B3] Cozzaglio L Coladonato M Doci R Travaglini P Vizzotto L Osio M Gennari L Horner’s syndrome as a complication of thyroidectomy: report of a case Surg Today 2008 11 1114 1116 10.1007/s00595-007-3741-z 19039637

[B4] Spinelli C Domini R, Miccoli P, Federici S, Spinelli C Tecniche e complicanze della chirurgia tiroidea Endocrinopatie pediatriche di interesse chirurgico 2000 Padova: Piccin 161 172

[B5] Kıray A Arman C Naderi S Guvencer M Korman E Surgical anatomy of the cervical sympathetic trunk Clin Anat 2005 11 179 185 10.1002/ca.20055 15768422

[B6] El-Labban GM Minimally invasive video-assisted thyroidectomy versus conventional thyroidectomy: a single-blinded, randomized controlled clinical trial J Minim Access Surg 2009 11 97 102 10.4103/0972-9941.59307 20407568 PMC2843132

[B7] Lombardi CP Raffaelli M De Crea C D’amore A Bellantone R Video-assisted thyroidectomy: lessons learned after more than one decade Acta Otorhinolaryngol Ital 2009 11 317 320 20463836 PMC2868209

[B8] Nordenström E Hallén M Nordenström J Horner syndrome is a serious complication in thyroid surgery: dissection in nerve stimulation may be a risk factor, shown in three cases Lakartidningen 2011 11 2660 2661 22474783

[B9] de Silva WD de Soysa MS Perera BL Iatrogenic Horner’s syndrome: a rare complication of thyroid surgery Ceylon Med J 2010 11 136 21341635 10.4038/cmj.v55i4.2639

[B10] Italiano D Cammaroto S Cedro C Bramanti P Ferlazzo E Horner syndrome following thyroidectomy Neurol Sci 2011 11 531 10.1007/s10072-010-0451-x 21088976

